# The Far East taiga forest: unrecognized inhospitable terrain for migrating Arctic-nesting waterbirds?

**DOI:** 10.7717/peerj.4353

**Published:** 2018-02-20

**Authors:** Xin Wang, Lei Cao, Inga Bysykatova, Zhenggang Xu, Sonia Rozenfeld, Wooseog Jeong, Didier Vangeluwe, Yunlin Zhao, Tianhe Xie, Kunpeng Yi, Anthony David Fox

**Affiliations:** 1 State Key Laboratory of Urban and Regional Ecology, Research Center for Eco-Environmental Sciences, Chinese Academy of Sciences, Beijing, China; 2 University of Chinese Academy of Sciences, Beijing, China; 3 Institute of Biological Problems of Cryolitozone, Siberian Branch of the Russian Academy of Sciences, Yakutsk, Russia; 4 Central South University of Forestry and Technology, Changsha, China; 5 Bird Ringing Centre of Russia, Institute of Ecology and Evolution, Russian Academy of Sciences, Moscow, Russian Federation; 6 Animal and Plant Quarantine Agency, Gimcheon, Republic of Korea; 7 Institut Royal des Sciences Naturelles de Belgique, Brussels, Belgium; 8 Department of Bioscience, Aarhus University, Aarhus, Denmark

**Keywords:** East Asian-Australasian Flyway, Ecological barrier, Geese, Satellite tracking, Siberian crane, Swans

## Abstract

The degree of inhospitable terrain encountered by migrating birds can dramatically affect migration strategies and their evolution as well as influence the way we develop our contemporary flyway conservation responses to protect them. We used telemetry data from 44 tagged individuals of four large-bodied, Arctic breeding waterbird species (two geese, a swan and one crane species) to show for the first time that these birds fly non-stop over the Far East taiga forest, despite their differing ecologies and migration routes. This implies a lack of suitable taiga refuelling habitats for these long-distance migrants. These results underline the extreme importance of northeast China spring staging habitats and of Arctic areas prior to departure in autumn to enable birds to clear this inhospitable biome, confirming the need for adequate site safeguard to protect these populations throughout their annual cycle.

## Introduction

Many migrating avian species undertake long uninterrupted flights across inhospitable terrain between breeding and wintering areas (Hahn et al., 2014; Henningsson & Alerstam, 2005), including the Pacific Ocean (Gill et al., 2009), the Himalayas (Bishop et al., 2015) and deserts (Ouwehand & Both, 2016). Large-bodied herbivorous geese wintering in Ireland and Britain breeding in Greenland cross 300–1,000 km of ocean between staging areas on Iceland where they regain depleted fuel for the journey (Weegman et al., 2017). Waterbirds crossing continents are assumed to put down and refuel on wetlands at will, because most studied western European, large-bodied waterbirds undertake relatively short migration episodes *en route* to and from continental arctic breeding areas (Eichhorn et al., 2009; Green et al., 2002; Van Wijk et al., 2012). Waterbirds breeding in high Arctic Far East Asia winter in the Chinese Yangtze River Floodplain and undertake a north-south migration route, traversing up to 2,500 km of continental boreal forest (“taiga”) after leaving NE China (Li, 2016), but we know nothing about how these birds might fly across this area. We fitted telemetry devices to four large-bodied, Arctic breeding waterbird species (two geese, one swan and one crane species) to analyse their migration/stopover patterns to compare with studies elsewhere.

## Materials & Methods

We captured 40 to 44 individuals of four large-bodied waterbird species on the wintering grounds or as flightless moulting adults or pre-flight juvenile geese, swans and cranes on the breeding grounds and fitted them with transmitters (Appendix A and see Yu et al. (2017) for full details of catch methods and devices). Bird capture and logger deployment were undertaken in accordance with the guidance and permission (No. rcees-ddll-001) of Research for Eco-Environmental Sciences, Chinese Academy of Sciences. We also reconstructed movement tracks of four Siberian cranes *Grus leucogeranus* reported by Li (2016) to generate georeferenced individual migration tracks entered into QGIS in comparable formats to our own data (Quantum GIS Development Team, 2017). Location and duration of individual stopping and staging were obtained from tables and maps in Li (2016). The duty cycle for generating GPS positional fixes differed between the model of transmitters and varied from one fix per hour to one fix per day, depending on transmitter type and battery condition. For movement data of Argos transmitters, we removed relocations with a duplicated timestamp and applied the algorithm of Douglas et al. (2012) to moderate location errors. These combined analyses generated 20 spring and 46 autumn migration episodes from the 44 different individual birds over two years (see the Appendix A). All migrations traversed the taiga forest. We only used complete migration tracks to compare with European populations.

We identified major staging areas by major clusters of sequential position data, which contrasted with consistent movements during flights (for details of the precise methods applied to define these, see Appendix B). This enabled identification of the arrival and departure dates and times (to the nearest 2 h) at major staging sites. From these, we were able to identify the timing and duration of stopovers and intervening migration flights, as well as the distances between stopovers. The differences in frequency of GPS positional fixes between transmitters did not affect results of the analyses, because inspection of the segmentation results showed that additional stopover sites could not have missed during any of the individual tracks (Fig. S1). We used *t*-tests to compare migration parameters of spring and autumn migrations.

We defined spring migration tracks within the period from day 40 to day 200 of the year and day 240 to 340 as autumn migration.

## Results

**Figure 1:**
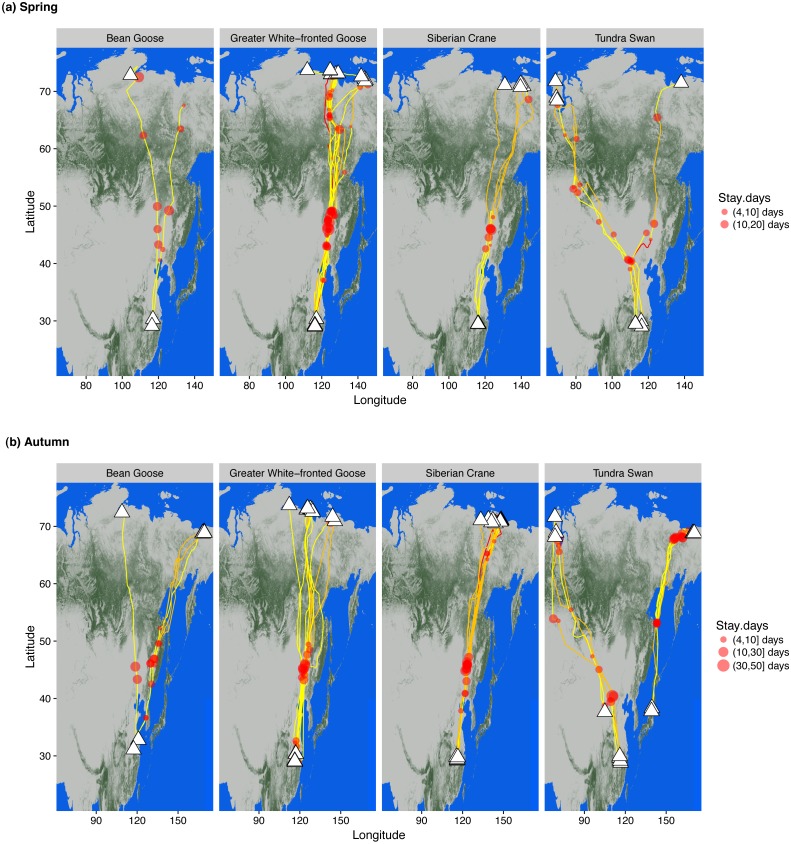
Wintering, staging and summering sites of bean and greater white-fronted geese, Siberian crane and tundra swan in (A) spring and (B) autumn. Individual routes describe tracks generated from GPS loggers attached to wild caught birds. White triangles represent ultimate wintering/summering sites. Red circle sizes indicate relative staging duration at each stopover site by each individual. Line colours indicate migration duration between adjacent staging/wintering/summering sites, yellow 0–5 days, orange 6–15 days and red 16–20 days. The degree of greenness on the map indicates the percentage of forest coverage derived from Hansen et al. (2013).

We tracked and obtained migration data from 15 tundra swans *Cygnus columbianus*, seven eastern tundra bean geese *Anser fabalis serrirostris*, nine greater white-fronted geese *A. albifrons* and 13 Siberian cranes instrumented with logger devices to track intra-annual movements throughout the annual cycle (Appendix A). The white-fronted geese and Siberian cranes partitioned their spring and autumn migrations into at least two major migration legs, both traversing the taiga forest ecoregion without stopping. Bean geese and tundra swans showed more frequent stops and shorter migration legs south of the taiga zone, but all four species flew over the taiga almost non-stop (Fig. 1). Only bean geese and greater white-fronted geese made short stopovers in a few wetlands along rivers within the taiga forest ecoregion. All staged in spring north of 60°N for periods of two weeks prior to arrival at ultimate breeding areas, providing breeding females with time to acquire fat and protein stores for reproductive investment prior to rapid follicular development before egg laying (Anderson et al., 2015).

Individual tundra swan migratory legs were longer (1,279.3 km ± 160.5 SE in spring and 1,944.6 ± 314.2 in autumn in the Far East Asia) than those of swans tracked in Europe (557–624 km and 1,032–1,142 km respectively (Beekman, Nolet & Klaassen, 2002; Nuijten et al., 2014), Table 1). Likewise, European greater white-fronted geese migrated in spring in legs of mean length 404 km with an average of 10 stopovers *en route* (Van Wijk et al., 2012) compared to 991.1 km (±106.0 SE) and an average of 5.6 stopovers in this study. Far East spring migration involved significantly more legs of significantly shorter distance (Table 1).

**Table 1:** Summary of migration legs in complete spring and autumn migrations.

Species	Mean value ± SE (sample size) for spring migration	Mean value ± SE (sample size) for autumn migration	df	*t*	*P* value
**Number of migration legs**					
Siberian Crane	3.0 ± 0.6 (4)	2.1 ± 0.3 (9)	4.28	1.40	0.23
Bean Goose	6 (1)	3.7 ± 0.3 (3)			
Tundra Swan	5.0 ± 0.7 (5)	2.9 ± 0.3 (7)	5.10	2.84	0.04
Greater White-fronted Goose	5.6 ± 0.3 (12)	2.9 ± 0.3 (11)	20.99	5.81	<0.01
**Length of migration leg (km)**					
Siberian Crane	1,628.7 ± 324.6 (12)	2,117.8 ± 244.5 (19)	22.60	−1.20	0.24
Bean Goose	864.5 ± 210.2 (6)	1,479.7 ± 397.5 (3)	14.16	−1.37	0.19
Tundra Swan	1,279.3 ± 160.5 (25)	1,944.6 ± 314.2 (20)	28.66	−1.89	0.07
Greater White-fronted Goose	991.1 ± 106.0 (67)	1,757.8 ± 219.3 (32)	46.00	−3.15	<0.01

## Discussion

For the first time, these data show that, despite following different routes, all individuals of four different species of large-bodied waterbirds staged south of the taiga forest before non-stop flights over this biogeographical zone. Most spring staging occurred in the tundra zone before arrival to ultimate summering areas. In autumn, all birds again crossed the entire taiga non-stop. These patterns common to four species of differing ecologies suggest that the Far East Asian taiga constitutes unfavourable feeding habitat for these birds, necessitating specific migration and refuelling strategies to cross. These results underline the extreme importance of northeast China spring staging habitats for accumulating body stores prior to the long flight to summering areas and of Arctic areas prior to departure in autumn, confirming the need for adequate site management to protect these populations throughout their annual cycle.

Theory predicts migrants should shorten spring migration, minimize number of stopovers and maximize migration leg distance, to arrive earliest at breeding areas (time-minimization strategy, Kokko, 1999; Moore, Smith & Sandberg, 2005); but autumn migrants have less time pressure and would increase numbers of stopovers and reduce migration leg distance (energy-minimization strategy, Nilsson, Klaassen & Alerstam, 2013; Zhao et al., 2017). Contrary to theoretical predictions, many of our tracked birds used more stopover sites and/or performed shorter migration legs in spring than in autumn. Despite differing diets, this is likely because all large-bodied waterbirds must acquire extra spring energy stores for migration (and ultimately for investment in reproduction) at successive stopover sites as these become successively freed by the variable timing of the vernal thaw. Following the Arctic summer pulse of biological productivity, waterbirds accumulate body stores close to breeding areas sufficient to move rapidly back to wintering grounds without needing to refuel before clearing the southern edge of the taiga. Future telemetry studies based on larger sample size will help answer these questions.

Why large herbivorous birds do not stage in the Far East taiga zone in spring and autumn as they do elsewhere remains unclear. The high (>1,000 m above sea level) altitude of the Far Eastern Asia taiga compared to that in western Eurasia is a potential explanation, delaying the spring thaw compared to lower altitudes (Appendix C). Whatever the reason, our results show the vital role of northeast China staging areas for all four species during both migration episodes for individuals wintering in the Yangtze River floodplain. Tundra swans breeding in the European and Far Eastern Russian tundra used more and different staging areas south of the taiga compared to the geese and cranes. Nevertheless, our results suggest that all four waterbird species are relatively robust to effects of climate change in the taiga, because the birds did not utilize wetlands in this area. However, reliance on staging areas in northeast China (which are subject to climate change and rapid anthropogenic change through economic development) and the Arctic (subject to more rapid climate change) underline the importance of maintaining viable habitats for wintering, spring- and autumn staging waterbirds along these parts of the existing flyway to maintain these populations. The maintenance of viable habitat is especially important because the Siberian crane remains critically endangered under IUCN Red List criteria and numbers of the other three taxa wintering in China are all currently declining (Jia et al., 2016).

## Conclusions

The 44 tagged individuals of four large-bodied, Arctic breeding waterbird species (two geese, a swan and one crane species) flew non-stop over the Far East taiga forest, despite their differing ecologies and migration routes. These results underline the extreme importance of northeast China spring staging habitats and of Arctic areas prior to departure in autumn to enable birds to clear this inhospitable biome, confirming the need for adequate site management to protect these populations throughout their annual cycle.

##  Supplemental Information

10.7717/peerj.4353/supp-1 Supplemental Information 1 Supplementary Material Appendix A. Details of tracked birds and loggers.
Appendix B. Detailed methods used to identify waterbird stopovers and flight segments.
Appendix C. Relief map showing the relative elevated height above sea level of the taiga zone of Far Eastern Asia compared to that of Western Eurasia.

## Additional Information and Declarations

### Data Availability

The raw data for this article was presented for review but cannot be published because waterbirds in the East Asian-Australasian Flyway are highly threatened by illegal hunting and open access of the tracking data will show the critical location and timing of stopovers, which could potentially impact upon the birds greatly in this instance.

## References

[ref-1] Anderson HB, Hubner CE, Speed JDM, Madsen J, Van der Wal R (2015). Biding time before breeding: flexible use of the Arctic landscape by migratory geese during spring. Polar Research.

[ref-2] Beekman JH, Nolet BA, Klaassen M (2002). Skipping swans: fuelling rates and wind conditions determine differential use of migratory stopover sites of Bewick’s Swans *Cygnus bewickii*. Ardea.

[ref-3] Bishop CM, Spivey RJ, Hawkes LA, Batbayar N, Chua B, Frappell PB, Milsom WK, Natsagdorj T, Newman SH, Scott GR, Takekawa JY, Wikelski M, Butler PJ (2015). The roller coaster flight strategy of bar-headed geese conserves energy during Himalayan migrations. Science.

[ref-4] Douglas DC, Weinzierl R, Davidson SC, Kays R, Wikelski M, Bohrer G (2012). Moderating Argos location errors in animal tracking data. Methods in Ecology and Evolution.

[ref-5] Eichhorn G, Drent RH, Stahl J, Leito A, Alerstam T (2009). Skipping the Baltic: the emergence of a dichotomy of alternative spring migration strategies in Russian barnacle geese. Journal of Animal Ecology.

[ref-6] Gill RE, Tibbitts TL, Douglas DC, Handel CM, Mulcahy DM, Gottschalck JC, Warnock N, McCaffery BJ, Battley PF, Piersma T (2009). Extreme endurance flights by landbirds crossing the Pacific Ocean: ecological corridor rather than barrier?. Proceedings of the Royal Society B: Biological Sciences.

[ref-7] Green M, Alerstam T, Clausen P, Drent R, Ebbinge BS (2002). Dark-bellied Brent Geese *Branta bernicla bernicla*, as recorded by satellite telemetry, do not minimize flight distance during spring migration. Ibis.

[ref-8] Hahn S, Emmenegger T, Lisovski S, Amrhein V, Zehtindjiev P, Liechti F (2014). Variable detours in long-distance migration across ecological barriers and their relation to habitat availability at ground. Ecology and Evolution.

[ref-9] Hansen MC, Potapov PV, Moore R, Hancher M, Turubanova SA, Tyukavina A, Thau D, Stehman SV, Goetz SJ, Loveland TR, Kommareddy A, Egorov A, Chini L, Justice CO, Townshend JRG (2013). High-resolution global maps of 21st-century forest cover change. Science.

[ref-10] Henningsson SS, Alerstam T (2005). Barriers and distances as determinants for the evolution of bird migration links: the arctic shorebird system. Proceedings of the Royal Society B: Biological Sciences.

[ref-11] Jia Q, Koyama K, Choi C, Kim H, Cao L, Gao D, Liu G, Fox AD (2016). Population estimates and geographical distributions of swans and geese in East Asia based on counts during the non-breeding season. Bird Conservation International.

[ref-12] Kokko H (1999). Competition for early arrival in migratory birds. Journal of Animal Ecology.

[ref-13] Li X (2016). Analysis of migration route and stopover sites of Siberian Crane (*Grus leucogranus*) by satellite tracking MSc.

[ref-14] Moore FR, Smith RJ, Sandberg R, Greenberg R, Marra PP (2005). Stopover ecology of intercontinental migrants. Birds of two worlds: the ecology and evolution of migration.

[ref-15] Nilsson C, Klaassen RHG, Alerstam T (2013). Differences in speed and duration of bird migration between spring and autumn. The American Naturalist.

[ref-16] Nuijten RJM, Kolzsch A, Van Gils JA, Hoye BJ, Oosterbeek K, De Vries PP, Klaassen M, Nolet BA (2014). The exception to the rule: retreating ice front makes Bewick’s swans *Cygnus columbianus bewickii* migrate slower in spring than in autumn. Journal of Avian Biology.

[ref-17] Ouwehand J, Both C (2016). Alternate non-stop migration strategies of pied flycatchers to cross the Sahara desert. Biology Letters.

[ref-18] Quantum GIS Development Team (2017). Quantum GIS geographic information system. Open source geospatial foundation project. https://qgis.org/en/site/.

[ref-19] Van Wijk RE, Kolzsch A, Kruckenberg H, Ebbinge BS, Muskens GJDM, Nolet BA (2012). Individually tracked geese follow peaks of temperature acceleration during spring migration. Oikos.

[ref-20] Weegman MD, Bearhop S, Hilton GM, Walsh AJ, Griffin L, Resheff Y, Nathan R, Fox AD (2017). Using accelerometry to compare costs of extended migration in an arctic herbivore. Current Zoology.

[ref-21] Yu H, Wang X, Cao L, Zhang L, Jia Q, Lee H, Xu ZG, Liu GH, Xu WB, Hu BH, Fox AD (2017). Are declining populations of wild geese in China ‘prisoners’ of their natural habitats?. Current Biology.

[ref-22] Zhao M, Christie M, Coleman J, Hassell C, Gosbell K, Lisovski S, Minton C, Klaassen M (2017). Time versus energy minimization migration strategy varies with body size and season in long-distance migratory shorebirds. Movement Ecology.

