# Supplementary material for: The Far East taiga forest: unrecognized inhospitable terrain for migrating Arctic-nesting waterbirds?
Source: PeerJ. 2018 Feb 20;6:e4353. doi: 10.7717/peerj.4353 (PMC5824675; doi:10.7717/peerj.4353)
Supplement: Supplemental Information 1 — Appendix A. Details of tracked birds and loggers. Appendix B. Detailed methods used to identify waterbird stopovers and flight segments. Appendix C. Relief map showing the relative elevated height above sea level of the taiga zone of Far Eastern Asia compared to that of Western Eurasia. [file peerj-06-4353-s001.docx]

**Supplementary Material**

Appendix A. Details of tracked birds and loggers.

| **Species** | **Transmitter model** | **Device**  **mass (g)** | **Type of telemetry device** | **Deployment type** | **Logger code**  **identifier** | **Spring**  **migration year** | **Autumn**  **migration year** | **Capture Location** | **Capture**  **month** | **Capture**  **method** |
| --- | --- | --- | --- | --- | --- | --- | --- | --- | --- | --- |
| Bean Goose | ECOTONE IBIS | 44 | GPS-GSM | Collar | APCN03 | 2016 | 2016 | Anhui Lakes, China | Nov, 2015 | Roost netting |
| Bean Goose | Druid DEBUT-45 | 45 | GPS-GSM | Collar | D40003c |  | 2016 | Lena River Delta, Russia | Aug, 2016 | Moult roundup |
| Bean Goose | Druid DEBUT-45 | 45 | GPS-GSM | Collar | D440040 |  | 2016 | Lena River Delta, Russia | Aug, 2016 | Moult roundup |
| Bean Goose | Druid DEBUT-45 | 45 | GPS-GSM | Collar | D440042 |  | 2016 | Lena River Delta, Russia | Aug, 2016 | Moult roundup |
| Bean Goose | Druid DEBUT-45 | 45 | GPS-GSM | Collar | D450046 |  | 2016 | Lena River Delta, Russia | Aug, 2016 | Moult roundup |
| Bean Goose | Druid DEBUT-45 | 45 | GPS-GSM | Collar | D450056 |  | 2016 | Lena River Delta, Russia | Aug, 2016 | Moult roundup |
| Bean Goose | KoEco Backpack | 50 | GPS-GSM | Backpack | vetch1504 | 2016 |  | Poyang Lake, China | Dec, 2015 | Lamping |
| Greater White-fronted Goose | HQXS BP3527 | 27 | GPS-GSM | Backpack | CAS002 | 2015 | 2015, 2016 | Poyang Lake, China | Jan, 2015 | Lamping |
| Greater White-fronted Goose | HQXS BP3527 | 27 | GPS-GSM | Backpack | CAS090 | 2016 | 2016 | Anhui Lakes, China | Oct, 2015 | Roost netting |
| Greater White-fronted Goose | HQXS BP3527 | 27 | GPS-GSM | Backpack | CAS092 | 2016 | 2016 | Anhui Lakes, China | Oct, 2015 | Roost netting |
| Greater White-fronted Goose | HQXS BP3527 | 27 | GPS-GSM | Backpack | CAS096 | 2016 | 2016 | Anhui Lakes, China | Oct, 2015 | Roost netting |
| Greater White-fronted Goose | HQXS BP3527 | 27 | GPS-GSM | Backpack | CAS097 | 2016 | 2016 | Anhui Lakes, China | Oct, 2015 | Roost netting |
| Greater White-fronted Goose | HQXS HQNN | 26 | GPS-GSM | Collar | CAS221 | 2016 |  | Poyang Lake, China | Dec, 2015 | Lamping |
| Greater White-fronted Goose | HQXS HQNN | 26 | GPS-GSM | Collar | CAS234 | 2016 | 2016 | Poyang Lake, China | Dec, 2015 | Lamping |
| Greater White-fronted Goose | HQXS BP3527 | 27 | GPS-GSM | Backpack | BYH024 | 2015 | 2015, 2016 | Poyang Lake, China | Dec, 2014 | Lamping |
| Greater White-fronted Goose | HQXS BP3527 | 27 | GPS-GSM | Backpack | BYH027 | 2015 | 2015, 2016 | Poyang Lake, China | Dec, 2014 | Lamping |
| Tundra Swan | ECOTONE IBIS | 35 | GPS-GSM | Collar | BSAK27 | 2016 | 2015, 2016 | Yamal Peninsula, Russia | Aug, 2015 | Moult roundup |
| Tundra Swan | ECOTONE IBIS | 35 | GPS-GSM | Collar | BSAK30 | 2016 | 2015, 2016 | Yamal Peninsula, Russia | Aug, 2015 | Moult roundup |
| Tundra Swan | ECOTONE IBIS | 35 | GPS-GSM | Collar | BSAK34 |  | 2016 | Yamal Peninsula, Russia | Aug, 2016 | Moult roundup |
| Tundra Swan | Druid DEBUT-45 | 45 | GPS-GSM | Collar | D2d0044 |  | 2016 | Lena River Delta, Russia | Aug, 2016 | Moult roundup |
| Tundra Swan | Druid DEBUT-45 | 45 | GPS-GSM | Collar | D400040 |  | 2016 | Lena River Delta, Russia | Aug, 2016 | Moult roundup |
| Tundra Swan | Druid DEBUT-45 | 45 | GPS-GSM | Collar | D400063 |  | 2016 | Lena River Delta, Russia | Aug, 2016 | Moult roundup |
| Tundra Swan | Druid DEBUT-45 | 45 | GPS-GSM | Collar | D44003b |  | 2016 | Lena River Delta, Russia | Aug, 2016 | Moult roundup |
| Tundra Swan | Druid DEBUT-45 | 45 | GPS-GSM | Collar | D440049 |  | 2016 | Lena River Delta, Russia | Aug, 2016 | Moult roundup |
| Tundra Swan | Druid DEBUT-45 | 45 | GPS-GSM | Collar | D45001e |  | 2016 | Lena River Delta, Russia | Aug, 2016 | Moult roundup |
| Tundra Swan | Druid DEBUT-45 | 45 | GPS-GSM | Collar | D450026 |  | 2016 | Lena River Delta, Russia | Aug, 2016 | Moult roundup |
| Tundra Swan | Druid DEBUT-45 | 45 | GPS-GSM | Collar | D450061 |  | 2016 | Lena River Delta, Russia | Aug, 2016 | Moult roundup |
| Tundra Swan | Druid DEBUT-45 | 45 | GPS-GSM | Collar | D450063 |  | 2016 | Lena River Delta, Russia | Aug, 2016 | Moult roundup |
| Tundra Swan | Druid DEBUT-45 | 45 | GPS-GSM | Collar | D45006d |  | 2016 | Lena River Delta, Russia | Aug, 2016 | Moult roundup |
| Tundra Swan | HQXS BP3527 | 27 | GPS-GSM | Backpack | BYH004 | 2015, 2016 |  | Dongting Lake, China | Jan, 2015 | Lamping |
| Tundra Swan | HQXS BP3527 | 27 | GPS-GSM | Backpack | BYH007 | 2015 | 2015 | Dongting Lake, China | Jan, 2015 | Lamping |
| Siberian Crane | NTT T-2050 | 65 | Doppler-ARGOS | Backpack | 19312 |  | 1996 | Indigirka River, Russia | July, 1995 | Moult roundup |
| Siberian Crane | NTT T-2050 | 65 | Doppler-ARGOS | Backpack | 19314 |  | 1996 | Indigirka River, Russia | Aug, 1995 | Moult roundup |
| Siberian Crane | NTT T-2050 | 65 | Doppler-ARGOS | Backpack | 21420 |  | 1995 | Indigirka River, Russia | Aug, 1995 | Moult roundup |
| Siberian Crane | NTT T-2050 | 65 | Doppler-ARGOS | Backpack | 21627 |  | 1995 | Indigirka River, Russia | July, 1996 | Moult roundup |
| Siberian Crane | NTT T-2050 | 65 | Doppler-ARGOS | Backpack | 21628 |  | 1995 | Indigirka River, Russia | July, 1996 | Moult roundup |
| Siberian Crane | NTT T-2050 | 65 | Doppler-ARGOS | Backpack | 21629 |  | 1996 | Indigirka River, Russia | July, 1996 | Moult roundup |
| Siberian Crane | NTT T-2050 | 65 | Doppler-ARGOS | Backpack | 22446 |  | 1996 | Indigirka River, Russia | July, 1996 | Moult roundup |
| Siberian Crane | NTT T-2050 | 65 | Doppler-ARGOS | Backpack | 22447 |  | 1996 | Indigirka River, Russia | July, 1996 | Moult roundup |
| Siberian Crane | NTT T-2050 | 65 | Doppler-ARGOS | Backpack | 22452 |  | 1996 | Indigirka River, Russia | July, 1996 | Moult roundup |
| Siberian Crane * | HQBP3622 | 22 | GPS-GSM | Backpack | 951 | 2015 | 2015 | Xianghai, Jilin, China | Oct, 2014 | Caught by reserve staff |
| Siberian Crane * | HQBP3622 | 22 | GPS-GSM | Backpack | 953 | 2015 | 2015 | Momoge, Jilin, China | Oct, 2014 | Caught by reserve staff |
| Siberian Crane * | HQBP3622 | 22 | GPS-GSM | Backpack | 955 | 2015 | 2015 | Momoge, Jilin, China | Oct, 2014 | Caught by reserve staff |
| Siberian Crane * | HQBP3622 | 22 | GPS-GSM | Backpack | 956 | 2015 | 2015 | Poyang Lake, China | Feb, 2015 | Lamping |

*extracted from Li (2016).

Appendix B. Detailed methods used to identify waterbird stopovers and flight segments.

## Segmentation of movement tracks

We modified the methods of (Barraquand & Benhamou 2008; Lavielle 2005; Le Corre et al. 2014) to objectively achieve movement track segmentation based on changes in movement behavior using the first passage time (FPT), a secondary signal of movement tracks (Edelhoff et al. 2016), applying the penalized contrast method proposed by (Lavielle 2005). FPT estimates the minimum duration of an animal crossing a given radius along its path, a measure that is low when an organism is travelling, high when it resides within a restricted area. The shift between high (whilst on staging areas) and low FPT (observed during migration) can be used to identify the departure and arrival dates during bouts of long-distance migrations and to identify seasonal ranges (e.g. breeding, staging and wintering areas) and migration routes (Bailleul et al. 2012). We set the range of optimal radius selection for FPT as a geometric sequence including 500 terms from 2.5 km to 50 km. We applied this as a hierarchical segmentation process with up to 4 iterations for each segment or until no further segmentation was forthcoming (Lavielle 2005).

## Identification of migration/stopover periods and sites

Following identification of discrete segments in individual tracking data established above, the next stage is to identify individual migration/stopover periods and sites. This was achieved in three steps: (i) identifying either flight segments or non-flight segments for the whole movement track, (ii) identifying sedentary (stopover/wintering/summering) segments from non-flight segments, and (iii) determining all non-sedentary segments as migration segments.

(i) We calculated the duration and net squared displacement (NSD, i.e. the minimum distance between locations, Bunnefeld et al. 2011) between the first and last locations of each identified segment. We classified segments as flight segments if: (1) the ratio of the NSD to segment duration was >150 km day^-1^ and NSD >150 km, or (2) the ratio of the NSD to duration was >75 km day^-1^ and NSD larger than >300 km. Segments failing to meet these criteria were classified as non-flight segments. Following this procedure, all segments were identified as flight or non- flight segments, but it remained likely that some flight segments contained bouts of continuous flight segments alternating with non- flight segments that remained to be identified, necessitating more rigorous identification of sedentary staging periods within the flight segment bouts (see next step).

(ii) The identification of sedentary segments comprised three steps, as follows. Firstly (1) adjacent flight segments (if any) were combined. Then the algorithm considered if all adjacent non-flight segments corresponded to a whole non-migration segment group. This was achieved by testing to see if the whole non-migration segment group duration (2) exceeded 4 days and comprised more than 5 relocations. If so, its minimum convex polygon (MCP) was calculated as an estimate of home range (Mohr 1947). If the MCP area <2,000 km^2^, (3) that combined non-migration segment group was identified as one sedentary segment. If the MCP >2,000 km^2^, the segment with the largest NSD between its first and last relocations (NSD_seg hereafter) was determined and the algorithm split the non-migration segment group to two sub-groups on the basis of the NSD_seg. Procedures (2) and (3) were repeated until all sedentary segments were identified, or the sub-groups could no longer be divided, that is, the sub-group comprised a single segment. From the sedentary segments we further identified wintering sites as those defined by last and first migration locations falling between day 320 and day 120 of the year and summering sites as those defined by last and first locations between day 180 and day 270 of the year.

(iii) Following identification of all sedentary segments in step (ii), we grouped all adjacent non-sedentary segments as migration segments, and calculated the distance and duration of the migration bouts.

All tracks are summarised in Fig. S1. An entire working flow diagram of the segmentation and identification procedure is illustrated in Fig. S2.

Figure S1: Summarised segmentation data of (a) spring and (b) autumn movement tracks from all individuals for all species, plotted as latitude against time (day of year).


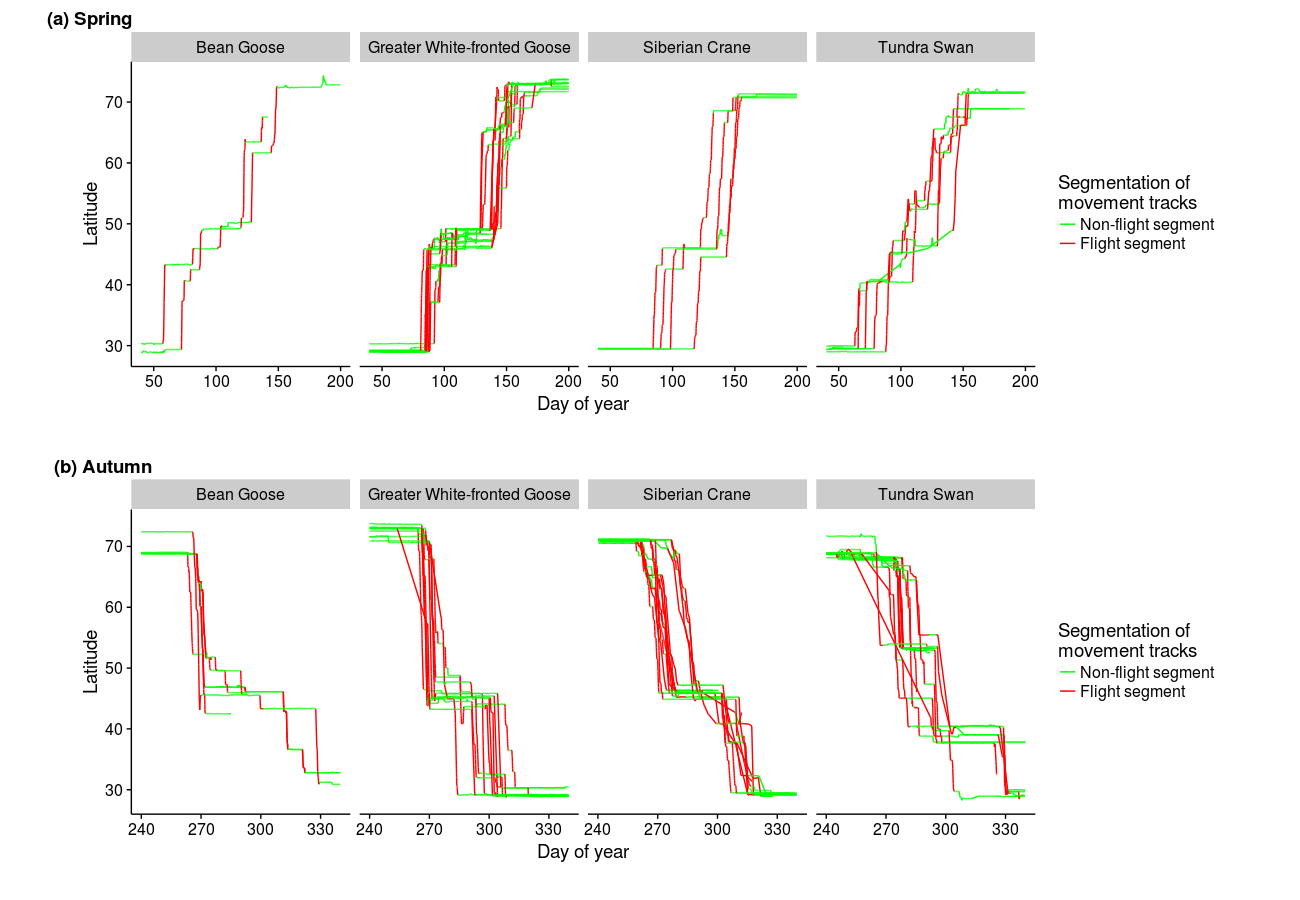


Figure S2: General working flow diagram of the process of segmentation and stopover site identification including levels to identify (i) flight segments, (ii) sedentary (stopover/wintering/summering) segments, and (iii) ultimately determining migration segments, see full description in the Material and methods in the main text.


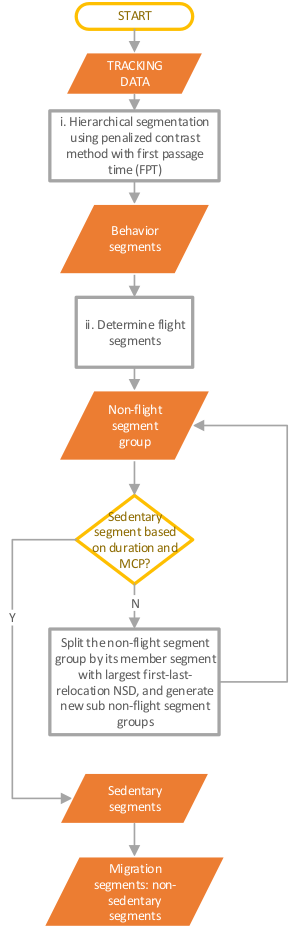


Appendix C. Relief map showing the relative elevated height above sea level of the taiga zone of Far Eastern Asia compared to that of Western Eurasia.


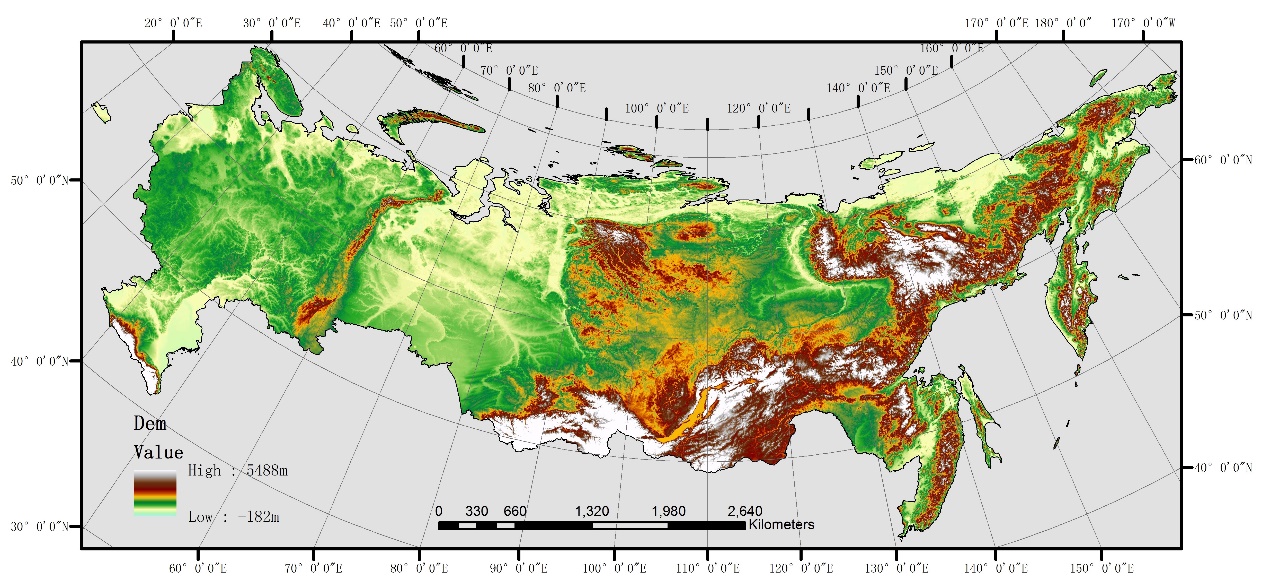


**References**

Bailleul F, Lesage V, Power M, Doidge DW, and Hammill MO. 2012. Migration phenology of beluga whales in a changing Arctic. *Climate Research* 53:169-178. 10.3354/cr01104

Barraquand F, and Benhamou S. 2008. Animal movements in heterogeneous landscapes: identifying profitable places and homogeneous movement bouts. *Ecology* 89. 10.1890/08-0162.1

Bunnefeld N, Boerger L, van Moorter B, Rolandsen CM, Dettki H, Solberg EJ, and Ericsson G. 2011. A model-driven approach to quantify migration patterns: individual, regional and yearly differences. *Journal of Animal Ecology* 80. 10.1111/j.1365-2656.2010.01776.x

Edelhoff H, Signer J, and Balkenhol N. 2016. Path segmentation for beginners: an overview of current methods for detecting changes in animal movement patterns. *Movement Ecology* 4:21. 10.1186/s40462-016-0086-5

Lavielle M. 2005. Using penalized contrasts for the change-point problem. *Signal Process* 85. 10.1016/j.sigpro.2005.01.012

Le Corre M, Dussault C, and Côté SD. 2014. Detecting changes in the annual movements of terrestrial migratory species: using the first-passage time to document the spring migration of caribou. *Movement Ecology* 2:19. 10.1186/s40462-014-0019-0

Li X. 2016. Analysis of migration route and stopover sites of Siberian Crane (*Grus leucogranus*) by satellite tracking MSc. Chinese Academy of Forestry.

Mohr CO. 1947. Table of equivalent populations of north American small mammals. *American Midland Naturalist* 37. 10.2307/2421652
